# Characteristics of *Streptococcus pneumoniae* Strains Colonizing Upper Respiratory Tract of Healthy Preschool Children in Poland

**DOI:** 10.1100/2012/732901

**Published:** 2012-08-02

**Authors:** Izabela Korona-Glowniak, Anna Malm

**Affiliations:** Department of Pharmaceutical Microbiology, Medical University of Lublin, Chodzki 1, 20-093 Lublin, Poland

## Abstract

Antibiotic resistant and invasive pneumococci may spread temporally and locally in day care centers (DCCs). We examined 267 children attending four DCCs located in the same city and 70 children staying at home in three seasons (autumn, winter, and spring) to determine prevalence, serotype distribution, antibiotic resistance patterns, and transmission of pneumococcal strains colonizing upper respiratory tract of healthy children without antipneumococcal vaccination. By pheno- and genotyping, we determined clonality of pneumococci, including drug-resistant strains. The average carriage of pneumococci in three seasons was 38.2%. 73.4% and 80.4% of the isolates belonged to serotypes present in 10- and 13-valent conjugate vaccine, respectively. Among the pneumococcal strains, 33.3% were susceptible to all antimicrobial tested and 39.2% had decreased susceptibility to penicillin. Multidrug resistance was common (35.7%); 97.5% of drug-resistant isolates represented serotypes included to 10- and 13-valent conjugate vaccine. According to BOX-PCR, clonality definitely was observed only in case of serotype 14. Multivariate analysis determined DCC attendance as strongly related to pneumococcal colonization in all three seasons, but important seasonal differences were demonstrated. In children attending DCCs, we observed dynamic turnover of pneumococcal strains, especially penicillin nonsusceptible and multidrug resistant, which were mostly distributed among serotypes included to available pneumococcal conjugate vaccines.

## 1. Introduction

Pneumococci are widely spread in the community, and they are a major etiologic agent of childhood bacteremia, meningitis, otitis media, pneumonia, and sinusitis [1, 2]. Carriage rates are particularly high in children attending day care centers (DCCs), and nasopharyngeal colonization is a major factor in horizontal transmission of pneumococcal disease, especially in this group of children [2, 3]. Only a small percentage of the colonized children will develop an infection, but pneumococcal nasopharyngeal isolates reflect the strains circulating the community and may predict the serotype of pneumococci causing invasive disease [4]. Recently, the situation aggravated worldwide due to the appearance and spread of pneumococcal strains that have acquired resistance to several classes of antimicrobials in commonly used in antipneumococcal therapy [5]. Due to geographical diversity of resistance of the *S. pneumoniae* population dependent on the local antimicrobial policy, epidemiological studies in each geographical region should be determined separately. In many countries, including Poland, the appearance and spreading of multidrug-resistant strains (MDR) was also observed [6].

Routine immunization with the pneumococcal conjugate vaccine (PCV) has been shown to decrease the incidence of vaccine-type antibiotic-resistant pneumococci both in invasive diseases and nasopharyngeal colonization [2]. Because of geographic variations in serotypes and drug-resistant isolates, a clear picture of the distribution of serotype associated with infection and colonization in various geographical areas is needed before launching of mass vaccination with conjugate vaccine.

It was previously observed that different pneumococcal serotypes or strains may dominate temporally and locally in different day care facilities [7–9]. In present study, we examined children attending four DCCs located in 3 different quarters of the city, and 70 children not attending DCC, staying at home, in three seasons (autumn, winter, and spring) to determine prevalence, serotype distribution, antibiotic resistance patterns, and transmission of *S. pneumoniae* strains colonizing upper respiratory tract of healthy children not vaccinated against pneumococci, with the emphasis on children attending day care centers (DCCs). By pheno- and genotyping, we determined clonality of pneumococci, including drug-resistant strains.

## 2. Material and Methods

### 2.1. Child Population and Questionnaire

The study was carried out in Lublin, a town of 40,000 inhabitants, in southeast Poland and enrolled 344 healthy children, aged between 3 and 5 years, whose parents agreed to participate. Two hundred sixty-seven children were recruited from four day care centers (DCCs) in Lublin (85 from DCC1, 63 from DCC2, 44 from DCC3, 75 from DCC4). Seventy-seven children, not attending DCC (staying at home), were recruited from 3 primary health care practices in Lublin. Upper respiratory colonization of *S. pneumoniae* was studied in three periods: in November-December 2002 (autumn), February-March 2003 (winter), and May-June 2003 (spring). Children who were absent on the day of sampling in one of the seasons because of prolonged illness were excluded. Finally, a total of 311 healthy children, with three times swabbing, were included in this study: 241 children who attended four DCCs (73 persons from DCC1, 58 from DCC2, 40 from DCC3, 70 from DCC4) and 70 children staying at home. Samples were collected at DCCs and primary health-care practices. At the time of first sampling, the parents were asked to fill in a questionnaire about individual children: age, gender, sibling (number and age), DCC attendance, passive smoking, medical history during preceding 3 months (number and type of illnesses), antibiotic consumption during preceding 2 months (number of completed antibiotic courses and type of antibiotic). A shorter questionnaire was administered at two ensuing visits to gather information on children illnesses and antibiotic consumption. Written informed consent was obtained from a parent/guardian of all studied children prior to the enrolment. None of the children were immunized by a pneumococcal vaccine.

### 2.2. Laboratory Procedures

Swabs from nostrils and throat were plated onto selective Mueller-Hinton agar with 5% sheep blood and 5 mg/L gentamicin and incubated aerobically at 35°C in a CO_2_-enriched atmosphere for 24–48 h. The *α*-hemolytic colonies exhibiting morphology suggestive of *S. pneumoniae* were isolated. Identification of these isolates was confirmed by susceptibility to optochin, bile solubility, and slide agglutination test (Slidex PneumoKit, BioMerieux). One colony per plate was then subcultured, harvested, and kept frozen at −70°C for further testing.

Susceptibility of isolates to antibiotics was determined by the disk diffusion method of Bauer and Kirby. Results were interpreted according to the European Committee on Antimicrobial Susceptibility Testing recommendations (EUCAST, 2011). Isolates exhibiting a zone of ≥20 mm around a 1 *μ*g oxacillin disk were reported as penicillin susceptible *S. pneumoniae* (PSSP); isolates exhibiting a zone of <20 mm were further tested by the *E*-test (AB Biodisk, Sweden), following the manufacturer's instruction, to determine minimal inhibitory concentration (MIC) for benzylpenicillin. Isolates with MIC ≤0.064 mg/L were considered as fully susceptible to benzylpenicillin; isolates with MIC >0.064 mg/L were called penicillin nonsusceptible *S. pneumoniae* (PNSSP). *S. pneumoniae* ATCC 49619 was used as control strain in the antimicrobial susceptibility tests. Phenotypic characterization of macrolide resistance (the constitutive-cMLSB; the partially inducible-iMcLSB; the inducible iMLSB; or the efflux-mediated—M) was determined on the basis of triple-disc test (erythromycin, clindamycin, and rokitamycin-ECRTD test) [10]. Multidrug-resistant (MDR-SP) isolates were defined as having resistance to at least 3 different classes of antibiotics.

Pneumococci were serotyped on the basis of capsular swelling (Quellung reaction) using antisera from the Statens Serum Institute (Copenhagen, Denmark). We applied antisera for determination of serotypes belonging to the 23-valent pneumococcal polysaccharide vaccine. The isolates negative to possessed pooled sera but positive to omni serum were defined as others.

### 2.3. BOX PCR Fingerprinting and Computer-Assisted Analysis

From 24 to 48 h *S. pneumoniae* cultures in Todd-Hewitt broth, the genomic DNA has been isolated using Genomic DNA Isolation and Purification Kit (Fermentas, Lithuania). DNA amplifications were performed according to van Belkum et al.  [11] using primer boxA. DNA banding patterns were analyzed using BIO-GENE analysis software according to the instruction of the manufacturer. A band tolerance setting of 1.7% was applied. A homology level of at least 95% was set as the definition of a separate genotype.

### 2.4. Statistical Analysis

Data processing and analysis were performed using StatSoft, Inc. STATISTICA 10. The potential predictor variables were tested in separate univariate analyses (Chi-squared or the Fisher exact test, as appropriate) for their association with upper respiratory colonization by *S. pneumoniae* in each season (autumn, winter, spring). Odds ratio (OR) and their 95% confidence intervals (CI) were calculated. Statistical significance was set at *P* < 0.05. Significant univariate predictors (*P* < 0.1) were tested for inclusion in the multivariate models. A model including all such predictors was constructed for each season, and nonsignificant variables were removed sequentially until only those significant at *P* < 0.1 remained. Variables of particular interest based on previous studies, such as children age, recent RTIs, and antibiotic use, were included even when were not statistically significant. Results of logistic regression analysis are reported as adjusted odds ratio (OR) with 95% CI. Statistical significance was set at *P* < 0.05.

## 3. Results

### 3.1. Upper Respiratory Colonization by *S. pneumoniae* and the Affecting Factors

During the study period, from November 2002 to June 2003, 933 nasopharyngeal samples were obtained in three seasons. A total of 311 children aged 3 to 5 were included in this study: 241 children attending four DCCs and 70 children staying at home. Demographic data of the studied children are given in Table 1. Of 356 positive pneumococcal cultures, 128 (36%) isolates were obtained from throat, 121 (34%) from nostrils, and 107 (30%) isolates colonized both throat and nostrils. *S. pneumoniae* (SP) was isolated from 115 (37%), 103 (33.1%), and 138 (44.4%) children in autumn, winter, and spring, respectively. Spring, as compared to winter, was a statistically significant factor associated with upper respiratory colonization by *S. pneumoniae* (*P* = 0.0051, OR 1.6, 95% CI 1.2–2.2). The average carriage rate among children attending DDCs was 41.9%, 40.2%, and 48.1% in autumn, winter, and spring, respectively. In contrast, the carriage rate among children staying at home was 20%, 24.3%, and 31,4% in these seasons, respectively.

Multivariate analysis determined DCC attendance as strongly related to SP colonization in all three seasons, but important seasonal differences in SP colonization were demonstrated (Table 2). DCC attendance and type of antibiotic used were independent predictors of SP colonization in autumn. Univariate analysis showed that, in this season, antibiotic consumption increased the likelihood of *S. pneumoniae* isolation (*P* = 0.04, OR 1.6, 95% CI 1.0–2.6), demonstrating significantly higher pneumococcal colonization among children after two antibiotic courses (*P* = 0.007, OR 1.8, 95% CI 1.3–2.7), and also among children who underwent treatment by *β*-lactam plus macrolide (*P* = 0.02, OR 2.1, 95% CI 1.3–3.3) in comparison to children without antibiotic therapy. According to multivariate analysis, DCC attendance, frequent respiratory tract infections (RTIs), and lower number of antibiotic courses increased the carriage rates significantly in winter. The rate of SP colonization in spring was increased independently by DCC attendance and younger age of the children.

### 3.2. Pneumococcal Serotypes

The 376  pneumococcal isolates were obtained from 356 positive samples: a single isolate was identified in 336 individuals, and 2 different isolates were found in 20 children. Genotypic analysis of isolates obtained from the same child in different seasons revealed that, in 30 cases of twice isolation and in 2 case of thrice isolation, the isolates were identical. Finally of the 342 tested isolates (293 strains isolated from DCC group and 49 strains isolated from home group), the most frequently prevalent serotypes were 6B (17.5%), 14 (13.7%), 19F (24.3%), and 23F (11.4%). 92.7% of isolates belonged to serotypes included in the 23-valent polysaccharide vaccine. The average prevalence of serotypes included in PCV10 (containing serotypes 1, 4, 5, 6B, 7F, 9V, 14, 18C, 19F, 23F) and PCV13 (containing serotypes 3, 6A, 19A additionally to 10-valent vaccine) was 73.4% and 80.4%, respectively. We observed the prevalence of specific serotypes in particular DCC—serotypes 15 and 18C were presented in DCC1 only, serotype 11A in DCC1 and DCC3, while serotype 9V was observed mainly in DCC4 (Table 3). More differential serotypes of pneumococci were isolated in home group in comparison to DCC groups.

### 3.3. Antibiotic Resistance among *S. pneumoniae* Isolates

Among the pneumococcal strains obtained in each of seasons, 33.3% were susceptible to all antimicrobial tested (31% in DCC group and 47% in home group) while 39.2% had decreased susceptibility to penicillin (MIC range 0.1–2.0 mg/L, MIC_50_ and MIC_90_ 1.0 mg/L). The tested pneumococci were resistant to cotrimoxazole (54.4%), tetracycline (41.2%), erythromycin (28.9%), clindamycin (28.9%), and chloramphenicol (30.1%). None of the tested isolates was resistant to rifampicin and teicoplanin. Rates of resistance to antimicrobial agents were higher among isolates recovered from DCC group in comparison to home group isolates and statistically significant in case of PNSSP (*P* = 0.01, OR 2.5, 95% CI 1.2–5.1). On the basis of the erythromycin-clindamycin-rokitamycin triple-disk test, 78 of the 99 erythromycin resistant strains were assigned to the cMLSB phenotype and 21 were iMcLSB. Multidrug resistance was common (35.7% of all isolates) and higher in DCC group isolates than in home group isolates (40.6% versus 32.6%, resp.). Among MDR-SP, 61,9% were nonsusceptible to penicillin. PNSSP and MDR-SP strains were mostly distributed among PCV13 serotypes (98.5% and 97.5%, resp.).

### 3.4. Phenotypes and Genotypes of *S. pneumoniae* in Different DCC

Each strain was characterized phenotypically by serotype and drug resistance pattern. Table 3 present, dynamics of phenotype prevalence in DCCs in following seasons. Phenotypes 6B PECcTeCSxt, 9V PSxt, and 19F ECcTeCSxt were specific for DCC4, and they were isolated in each season. Phenotype 19F TeCSxt was found mostly in DCC2 in each season, such as 23F S. In DCC3 only, presence of 19F Sxt phenotype and most of strains with 23F TeSxt phenotype was observed. There were phenotypes widespread in more than one setting: 19F PECcTeCSxt was present in DCC1, DCC3, DCC4, and phenotype 14 P was observed in DCC1, DCC2, DCC3, while 14 PSxt was observed mainly in DCC4. Isolates with serotype 6B and susceptible to all tested antibiotic (6B S) were found only in children not attending to DCC.

Using BOX PCR technique, from 185 distinct BOX patterns, 54 groups of similarity were identified when 85% level of similarity was used for grouping the isolates and 36 isolates had unique genotype. Within groups of similarity, subtypes of genotypes were determined (Table 4). Strains with phenotype 19F PECcTeCSxt were isolated from DCC3 (genotypes 65, 67) and DCC4 (genotypes 19, 22, 35) where they appeared in close relation with 19F ECcTeCSxt strains (genotypes 36, 37). Genotype 64 consisted of 19F PECcTeCSxt strains that came from DCC1. Phenotype 19F TeCSxt was specific for DCC2, and these strains showed similarity (genotypes 71, 72). Pneumococci with 6B PECcTeCSxt phenotype derived from DCC4 were shared into three genotypes (11, 16, 17). Serotype 23F appeared the highest genetic discrimination despite connection specific phenotypes with given DCC (23F S-DCC2; 23F TeSxt-DCC3). However, clonality of strains definitely was observed only in case of serotype 14, because 48 isolates had 87% of similarity independent on resistance pattern and group of children from which they were isolated and independent on season.

## 4. Discussion

Our study contains the epidemiological characteristics of *S. pneumoniae* strains colonizing the upper respiratory tract among asymptomatic children in Poland, whose problem has been conducted in many other countries [12–15]. However, despite this and the advances in the development of pneumococcal conjugate vaccines (PCVs) leading to a reduction of invasive disorders, eradication of pneumococcal diseases is not within easy reach.

The nasopharyngeal carriage rates of *S. pneumoniae* reported worldwide range from 2.3% to 80% in various populations. The highest reported carriage rate was found in children under 3 years old [12, 16, 17]. Our data revealed a high frequency of upper respiratory colonization by pneumococci among healthy children aged 3–5, not vaccinated against pneumococci. When analyzing our data from each sampling period, the rate of *S. pneumoniae* presence in upper respiratory tract (ranging from 33 to 44%) was at similar level to those reported by other authors, involving the same age group and the same type of clinical specimen [15, 18].

Nasopharyngeal sampling is considered usually to be far better to oropharyngeal sampling for detecting *S. pneumoniae*, especially in young children [19], in adults oropharyngeal sampling yielded higher isolation rates [20]. However, Capeding et al. [21] showed in all age groups that *S. pneumoniae* was isolated significantly more often from the nasal site than from the oropharyngeal site. In our study, nasal and oropharyngeal colonization rates were comparable. Our data and those from literature suggested that the rate of nasopharyngeal, nasal, or oropharyngeal colonization of *S. pneumoniae* may be dependent on the studied population.

Different data regarding seasonal fluctuations in carriage of *S. pneumoniae* in infants and children were described: the decreased rate of colonization in healthy children between seasons [22], no seasonal fluctuations in the pneumococcal carriage [14], or the increase of pneumococcal carriage in the winter [23]. Moreover, Marchisio et al. [24] observed the increase in the proportion of children who were asymptomatic nasopharyngeal carriers of *S. pneumoniae* and H. influenzae between autumn and spring. In our study, spring appeared to be significantly favorable to colonization by *S. pneumoniae* in comparison to winter.

The data presented in our study seem to indicate that, in different seasons, the different factors were associated with pneumococcal colonization. However, we found that, irrespective of the season, DCC attendance was the strongest predictor of SP colonization. This observation was in accordance with data of other authors [3, 13, 15, 18, 23]. We observed that, in spring, the predictors of pneumococcal colonization were younger age and DCC attendance, whereas in winter frequent RTIs and antibiotic treatment. It is not surprising that in winter children have frequent RTIs, mainly of viral etiology, which may injure mucous membrane and cause damage of the local immunological host response facilitating adherence of bacteria [23, 25–27]. Syrjänen et al. [28] reported that nasopharyngeal carriage of pneumococci during RTIs (without otitis media) in children increased from 13–43% to 45–56%, depending on age. On the contrary, Greenberg et al. [20] found no differences in the overall *S. pneumoniae* carriage between healthy and sick children in different age groups.

It was found in this paper that frequent RTIs during the autumn and winter (above 60% children) were connected with antibiotic medication (45% and 42%, resp.) and longer absence in DCC. It may have resulted in gradual and transient elimination of colonizing *S. pneumoniae* in the population in winter, whereas pneumococci spread in the spring with superiority of PNSSP. Regev-Yochay et al. [29] described that a suppressive effect of antimicrobials on *S. pneumoniae* carriage rate was short (up to 1 month), while the opposite effect on PNSSP carriage lasted for 3 months.

The young children are the group the most often treated with antimicrobial drugs due to frequent RTIs. Up to 40% of the enrolled children had received one or more courses of antibiotic therapy in the previous 2 months in autumn and in winter. In the present study, antibiotic consumption in general appeared to be statistically significant factor associated with pneumococcal colonization in autumn. Borer et al. [25] and Katsarolis et al. [26] reported that the use of antibiotics caused the increase of carriage rate of *S. pneumoniae*, whereas Principi et al. [30] found macrolide, medication to be a risk factor for nasopharyngeal colonization of pneumococci. In our study, children, who underwent two antibiotic courses in the previous 2 months and were treated by *β*-lactam plus macrolide demonstrated significantly higher pneumococcal colonization. However, we showed that, in winter season children with higher number of antibiotic courses had a lower rate of *S. pneumoniae* colonization. This situation could be possible during antibiotic therapy or in short time after it, as reported by other authors. Such findings were described by Regev-Yochay et al. [29], Pebody et al. [13], and Greenberg et al. [20], who found that antibiotic treatment during one month before screening significantly lowered the *S. pneumoniae* carriage rate. This may be a consequence of the antibiotic abuse in autumn as well as absence of children in DCC due to illness before swabbing which could cause the lower rate of transmission of pneumococci from person to person and subsequently could reduce rate of colonization in winter.

Overuse of antibiotics in industrialized counties has contributed to an acceleration of the emergence and spread of microbial resistance. The percentage of PNSSP isolates has been reported to be 0–60% among healthy children in Europe [28, 30–33]. Data presented in this study confirmed that prevalence of PNSSP in Poland has been constantly increasing. In the recent Alexander Project covering years from 1998 to 2000, the frequency of PNSSP was 12.3% [34], but significant increase to 20.3% was observed among clinical isolates in 2002 [35]. However, in the same time in Poland, the percentage of PNSSP isolates from healthy children under 5 years in settings such as DCCs and orphanages was 36.2% [31], comparable to our data.

The prevalence of erythromycin resistance exceeded that of penicillin resistance in the majority of countries, but, in our study, resistance to penicillin was more common, comparable to average data obtained in East Europe during Alexander Project [34]. Macrolide resistance of pneumococci observed in present study was MLSB type only that is consistent with the results of other studies from European countries showing the predominance of this resistance mechanism [33, 36]. It has been known that coresistance to macrolide, lincosamide, and streptogramin B can be expressed either constitutively (cMLSB phenotype) or inducibly (iMLSB phenotype) [10]. In our study, by using erythromycin-clindamycin double disk test, conventionally used to identify three resistant phenotypes (cMLSB, iMLSB, M), all erythromycin-resistant pneumococcal strains were assigned to the cMLSB phenotype. ECRTD test allowed differentiation of these strains into cMLSB and iMcLSB phenotypes. These data confirmed that double disk test appears to be less applicable for *S. pneumoniae* strains because pneumococci with MLSB phenotype, carrying the erm(B) gene, are resistant to clindamycin without induction. Inducibility in pneumococci regards macrolides (particularly 16 membered ones, with emphasis on rokitamycin) but not lincosamides [10, 36].

Each DCC can be regarded as independent microenvironment responsible for the specific acquisition and spread of pneumococci [7, 9]. We found differences among the DCCs concerning most of the serotypes, antibiotic resistance, and genotypic patterns. A few serotypes (9V,18C, 15) were typical only for one DCC. We observed different levels of antibiotic resistance in each DCC: the highest in DCC4. After genetic analysis, it was shown that the differences depended on presence of particular strains circulating in single DCC in one or more seasons. Strains with the same phenotypes were homogenous within one DCC, but they yielded different BOX genotype for each DCC.

BOX-PCR method possesses high discriminate power, and, because of this capacity, it is useful for comparison many strains from the same population [37]. Several studies concerning analysis of *S. pneumoniae* isolated from healthy children attending DCCs have been done with applying molecular typing in other country [9, 32] and suggested that each DCC may be considered as an autonomous epidemiological unit where the epidemic resistant clones of *S. pneumoniae* appeared and spread. Data presented in this paper confirm that DCC environment facilitates person-to-person transmission of pneumococci, especially drug resistant strains, and may serve as a reservoir of drug-resistant strains, hence augmenting their carriage in the community.

The World Health Organization (WHO) considers immunization of infants and young children with pneumococcal vaccines a priority. Data published by other authors suggested that vaccination could potentially reduce the carriage rate of antibiotic-resistant pneumococci in Europe [31]. Currently, in Poland, both 23-valent polysaccharide vaccine for children ≥2 years old and adults (since 1996) as well as pneumococcal conjugate vaccines PCV7 (since 2006), PCV10 (since 2009), and PCV13 (since 2010) for children ≤2 years old have been recommended in the national immunization schedule. Since 2006, less than 5% of children ≤5 years were vaccinated per year, so currently we supposed that less than 20% of children in this group of age are immunized by PCV [38]. PCV7 is currently being replaced by PCV13 vaccine as manufacturing and supply is scaled up. Our data confirm information indicating higher effectiveness of new vaccines [39]. An encouraging finding was that a majority of PNSSP and MDR-SP belonged to serotypes included in PCV13 and PCV10, but there is no spectacular difference between covering of drug-resistant serotypes belonged to PCV7 and PCV10/PCV13.

Even though 25.4% children were colonized twice and 8.4% children were colonized thrice during our study, in most cases, pneumococci isolated from the same child in 2 different seasons were unrelated. Two children carried the same pneumococcal strain for more than 6 months (in three seasons of our study), and two children were colonized by identical strain in autumn and spring (without pneumococcal isolation in winter) probably due to too small number of bacteria in the throat/nose made isolation impossible. Moreover, 28 children were colonized by identical strain for 3 months. This confirmed high dynamics of pneumococcal strain replacement in short time among healthy preschool children attending DCC [31]. Prolonged pneumococcal colonization was observed by other authors [31, 32], and it has been inversely correlated with age but also depends on genetic backgrounds of both host and bacteria [40]. We did not observe correlation between prolonged colonization and age. However, 75.8% strains colonizing tested children up to 6 months belonged to 6B (24.2%), 14 (18.2%), and 19F (33.3%) serotypes which are known to be weak inducers of immunity [1, 27, 31, 32].

## 5. Conclusion

The present study demonstrated that upper respiratory colonization with *S. pneumoniae* in preschool children is a dynamic process. We found seasonal variations in the rate of pneumococcal colonization as well as in the factors affecting colonization. Autumn appears to be exceptional season when children meet each other in large groups after summer holidays and they have more RTIs due to seasonal collapsing of immunity and frequently received antibiotic therapy. In this season, DCC attendance and antibiotic therapy (number of courses and type of antibiotic) were the factors predisposing to SP colonization. In winter, continuation of antibiotic therapy causing cumulation of antibiotic courses probably combined with children absence in DCC due to infections were the factors which limited frequency of pneumococcal colonization in this season. In spring, children come back to DCC after illnesses and frequency of colonization increases. Our observation clearly indicated that each DCC is unique setting, which creates conditions conducive to the transmission of pneumococci, including drug-resistant strains, and the appearance and efficient spread of endemic/epidemic genotypes are strongly associated with greater antibiotic pressure and presence of greater group of children. The information that PNSSP and MDR-SP strains belonged to PCV10 and PCV13 serotypes may be stimulating to introduction of these vaccines to national vaccination program for all children aged 2–59 months which should prevent the carriage and spread of these strains in DCCs and have a positive impact on the development of herd immunity in Poland.

## Figures and Tables

**Table 1:** Demographic data of studied children.

		Number of children (%)					
Risk factor		DCC1	DCC2	DCC3	DCC4	Home	All
		(*n* = 73)	(*n* = 58)	(*n* = 40)	(*n* = 70)	(*n* = 70)	(*n* = 311)
Age (yr)	3	10 (13.7)	15 (25.9)	13 (32.5)	16 (22.9)	36 (51.4)	90 (28.9)
at entry	4	26 (35.6)	18 (31.0)	15 (37.5)	33 (47.1)	23 (32.9)	115 (37.0)
	5	37 (50.7)	25 (43.1)	12 (30.0)	21 (30.0)	11 (15.7)	106 (34.1)
Sex	Female	35 (48.0)	31 (53.5)	24 (60.0)	33 (47.1)	35 (50.0)	158 (50.8)
	Male	38 (52.0)	27 (46.6)	16 (40.0)	37 (52.9)	35 (50.0)	153 (49.2)
Sibling	No	25 (34.3)	27 (46.6)	27 (67.5)	36 (51.4)	20 (28.6)	135 (43.4)
possessing	1	41 (56.2)	21 (36.2)	12 (30.0)	30 (42.9)	35 (50.0)	139 (44.7)
	2	7 (9.6)	10 (17.2)	1 (2.5)	4 (5.7)	15 (21.4)	37 (11.9)
Siblings	≤2	5 (6.8)	3 (5.2)	3 (7.5)	5 (7.1)	9 (12.9)	25 (8.0)
age (yr)	3–5	6 (8.2)	0 (0)	0 (0)	2 (2.9)	13 (18.6)	21 (6.7)
	≥6	34 (46.6)	23 (40.0)	10 (25.0)	25 (35.7)	23 (32.9)	115 (37.0)
	≤2 and 3–5	1 (1.4)	0 (0)	0 (0)	0 (0)	2 (2.9)	3 (1.0)
	≤2 and ≥6	1 (1.4)	0 (0)	0 (0)	1 (1.4)	1 (1.4)	3 (1.0)
	3–5 and ≥6	0 (0)	5 (8.6)	0 (0)	1 (1.4)	2 (2.9)	8 (2.6)
	≤2 and 3–5 and ≥6	1 (1.4)	0 (0)	0 (0)	0 (0)	0 (0)	1 (0.3)
Passive smoking		31 (42.5)	16 (27.6)	13 (32.5)	34 (48.6)	22 (34.3)	118 (37.9)

		Autumn		Winter		Spring	

Antibiotic consumption^a^		141 (45.3)		132 (42.4)		105 (33.8)	
*β*-Lactam		73 (23.5)		76 (24.4)		61 (19.6)	
Macrolide		8 (2.6)		13 (4.2)		15 (4.8)	
Cotrimoxazole		19 (6.1)		9 (2.9)		8 (2.6)	
*β*-Lactam and macrolide		12 (3.8)		13 (4.2)		4 (1.3)	
*β*-Lactam and cootrimoxazole		6 (1.9)		4 (1.3)		3 (1.0)	
Macrolide and cotrimoxazole		1 (0.3)		1 (0.3)		0 (0)	
*β*-Lactam and macrolide and cotrimoxazole		3 (1.0)		0 (0)		0 (0)	
No data		14 (4.5)		12 (3.9)		8 (2.6)	
RTIs^b^		199 (64.0)		190 (61.1)		137 (44.1)	
Otitis media		13 (4.2)		15 (4.8)		8 (2.6)	
Pharyngitis		62 (19.9)		40 (12.9)		56 (18.0)	
Pneumonia		3 (1.0)		4 (1.3)		1 (0.3)	
Bronchitis		48 (15.4)		27 (8.7)		13 (4.2)	
Sinusitis		2 (0.6)		2 (0.6)		3 (1.0)	
Hospitalization^a^		14 (4.5)		6 (1.9)		7 (2.3)	

^a^in previous 2 months before swabbing; ^b^in previous 3 months before swabbing; RTIs: respiratory tract infections; DCC: day care center.

**Table 2:** Multivariate analysis of predictors of upper respiratory colonization of *S. pneumonia* in healthy preschool children in following seasons.

Predictors	Autumn		Winter		Spring	
	OR (95% CI)	*P*	OR (95% CI)	*P*	OR (95% CI)	*P*
Age			0.75 (0.5–1.0)	0.08	0.6 (0.5–0.8)	0.003
DCC attendance	2.5 (1.3–4.9)	0.005	2.1 (1.1–4.0)	0.02	2.6 (1.4–4.8)	0.001
RTIs			1.9 (1.1–3.5)	0.03		
Number of Ab courses			0.5 (0.3–0.8)	0.002		
Type of Ab	1.0	0.04				

DCC: day care center; RTIs: respiratory tract infections; Ab: antibiotic.

**Table 3:** Dynamics of phenotype prevalence of *S. pneumoniae* isolates in healthy preschool children in DCCs in following seasons.

		Number of isolates											
Phenotype			DCC1			DCC2			DCC3			DCC4	
		Autumn	Winter	Spring	Autumn	Winter	Spring	Autumn	Winter	Spring	Autumn	Winter	Spring
3	S	2	2	—	—	—	—	3	1	—	2	3	5
6A	S	—	—	—	—	—	—	—	—	1	—	—	—
6B	S	1	1	8	2	1	—	—	1	—	—	—	—
6B	PECcTeCSxt	1	1	—	—	—	—	—	—	—	9	9	6
6B	ECcTe	—	—	1	—	1	3	—	—	—	—	—	—
6B	ECcTeSxt	—	—	—	—	—	—	1	1	—	—	—	—
6B	PECcTeSxt	—	—	—	—	—	—	—	—	1	—	—	—
6B	PECcTe	1	—	—	4	2	—	1	—	—	—	—	—
7F	S	—	—	—	1	—	2	—	—	—	—	—	—
9V	PSxt	—	—	—	—	1	1	—	—	—	8	3	2
11A	S	4	2	4	—	—	—	3	1	3	—	—	—
14	P	2	3	4	—	4	6	—	1	8	—	—	—
14	PSxt	1	—	—	—	1	4	1	—	—	2	6	8
15B	S	3	—	—	—	—	—	—	—	—	—	—	—
15B	Sxt	3	2	3	—	—	—	—	—	—	—	—	—
15B	PSxt	1	—	—	—	—	—	—	—	—	—	—	—
18C	SXT	1	2	1	—	—	—	—	—	—	—	—	—
19F	PECcTeCSxt	4	2	7	—	—	—	1	7	8	6	4	1
19F	PECcTeC	1	—	—	—	—	—	—	—	—	—	—	—
19F	Sxt	—	—	—	—	—	—	—	1	5	—	—	—
19F	TeCSxt	—	—	—	7	3	8	2	—	1	—	—	—
19F	ECcTeCSxt	—	—	—	—	—	—	—	—	—	3	7	1
23A	S	—	—	1	—	—	1	1	—	—	—	—	—
23A	ECcTeCSxt	—	—	—	—	—	—	—	—	—	—	—	2
23F	S	—	—	—	3	4	6	—	—	—	—	—	—
23F	SXT	—	—	—	—	—	—	—	—	—	1	—	—
23F	ECcTe	—	—	—	1	—	—	—	—	—	—	—	—
23F	ECcTeCSxt	—	—	—	—	—	—	—	—	—	1	—	—
23F	TeSxt	2	—	1	—	1	—	3	2	3	1	1	—
23F	TeCSxt	—	—	—	—	—	—	—	2	1	—	—	—

DCC: day care center; P: penicillin, E: erythromycin; Cc: clindamycin; Te: tetracycline; C: chloramphenicol; Sxt: cotrimoxazol; S: sensitive to all tested antibiotics.

**Table 4:** BOX-PCR genotypes of *S. pneumoniae* isolates in healthy preschool children in DCCs and home group.

Genotypes^a^	Number of strains	Serotype	Antibiotic resistance (number of strains)	Number of strains				
				DCC1	DCC2	DCC3	DCC4	Home
**1**	6	11A	S	5				1
**2**	2	11A	S	2				
**3**	2	11A	S	2				
**4**	2	11A	S	1				1

**5**	2	11A	S			2		
**6**	5	11A	S			5		

**7**	48	14	P(28); PSxt (20)	9	14	10	15	
**8**	2	14	P					2
**10**	2	7F	S		2			
**11**	18	6B	PECcTeCSxt				18	

**16**	5	6B	PECcTeCSxt				5	
**17**	2	6B	PECcTeCSxt				2	

**18**	15	9V	PSxt		2		13	

**19**	7	19F	PECcTeCSxt				6	
		6B	S	1				
**20**	3	23F	TeSxt			3		
**21**	5	19F	PECcTeCSxt					5
**22**	3	19F	PECcTeCSxt (2); ECcTeCSxt (1)				3	
**23**	2	3	S			2		
**24**	4	6B	PECcTe		4			
**25**	3	6B	ECcTeCSxt (2); PECcTe(1)			3		
**27**	3	3	S	3				
**29**	6	6B	ECcTe(4); S(2)	1	5			
**30**	4	6B	S	4				
**31**	6	6B	S	5	1			
**32**	10	6B	S					10
**33**	3	6B	PECcTe(2); ECcTe(1)		3			
**35**	3	19F	PECcTeCSxt				3	
**36**	7	19F	ECcTeCSxt				7	
**37**	3	19F	ECcTeCSxt				3	

**38**	8	23F	ECcTeCSxt (1);				1	
			TeSxt (6); Sxt (1)			4	3	
**39**	2	23F	ECcTeCSxt (1);					1
			TeSxt (1)			1		
**40**	3	6B	PECcTeCSxt (2);	2				
			Sxt (1)			1		

**41**	2	18C	S(1); Sxt (1)	1				1
**42**	3	18C	Sxt	3				

**45**	6	15B	Sxt (5); PSxt (1)	6				

**47**	2	23F	S		2			
**50**	3	23F	TeCSxt			3		
**52**	2	23A	ECcTeCSxt				2	
**53**	2	23F	S	1	1			

**58**	5	19F	TeCSxt		5			

**60**	2	23F	ECcTeSxt (1);					1
		23F	TSXT(1)					1
**64**	14	19F	PECcTeCSxt	14				
**65**	9	19F	PECcTeCSxt			9		
**67**	6	19F	PECcTeCSxt			6		
**68**	3	3	S	1		2		
**69**	8	3	S			7		1

**70**	3	3	S			3		

**71**	9	19F	TeCSxt		9			
**72**	7	19F	TeCSxt		4	3		

**76**	4	19F	Sxt			4		
**77**	11	23F	S(10); TeSxt (1)		11			
**79**	4	23F	TeSxt (3); TeCSxt (1)	2				2

**81**	9	19F	TeCSxt (5);					5
			ECcTeSxt (2);					2
			ECcTeCSxt (2)					2

**85**	2	9N	S					2

^a^Strains with similarity factor >60% were grouped in one table line. DCC: day care center; P: penicillin; E: erythromycin; Cc: clindamycin; Te: tetracycline; C: chloramphenicol; Sxt: co-trimoxazole; S: sensitive to all tested antibiotics.
